# Effects of Frequency-Labeled Narrowband Noise on Postural Stability and Cortical Activation: An fNIRS Study

**DOI:** 10.3390/brainsci16070739

**Published:** 2026-07-12

**Authors:** Sang Seok Yeo, Zi Han Sun, Dong Hyun Byun

**Affiliations:** 1 Department of Physical Therapy, College of Health Sciences, Dankook University, 119, Dandae-ro, Dongnam-gu, Cheonan-si 31116, Republic of Korea; yeopt@dankook.ac.kr; 2 Department of Health, Graduate School, Dankook University, 119, Dandae-ro, Dongnam-gu, Cheonan-si 31116, Republic of Korea; sunzihan990224@naver.com; 3 Department of Special Creative Convergence, College of Rehabilitation Sciences, Daegu University, Daegu-daero 201, Jillyang-eup, Gyeongsan-si 38453, Republic of Korea

**Keywords:** narrowband noise, static balance, cortical activation, frequency bands, functional near-infrared spectroscopy

## Abstract

**Highlights:**

**What are the main findings?**

- Mid-frequency narrowband noise (2000 Hz) yields the most stable postural control during an eyes-closed tandem stance, significantly reducing sway length, ellipse surface, and average speed.
- High-frequency narrowband noise (5000 Hz) produces the greatest postural instability and elicits significantly higher bilateral activation in the premotor cortex (PMC) compared to low-frequency conditions.

**What are the implications of the main findings?**

- The frequency characteristics of narrowband noise differentially modulate static balance and corresponding cortical activation.
- The concurrent increase in postural instability and bilateral PMC activation under high-frequency stimulation suggests that specific auditory frequencies impose distinct neural demands during sensorimotor integration.

**Abstract:**

**Background:** Maintaining postural stability requires the integration of multisensory information, including visual, vestibular, and somatosensory inputs. This study aimed to investigate the effects of narrowband noise across different frequency bands on static postural balance and cortical activation in healthy adults. **Methods:** Twenty healthy adults participated in a repeated-measures experiment in which they maintained a tandem stance with eyes closed under four conditions: no sound, low-frequency narrowband noise (500 Hz), mid-frequency narrowband noise (2000 Hz), and high-frequency narrowband noise (5000 Hz). Static balance was assessed using center of pressure parameters, including sway length, ellipse surface, delta X, and delta Y, recorded via a pressure measurement platform. Cortical activation in the premotor cortex (PMC), frontal eye fields (FEF), superior temporal gyrus (STG), and middle temporal gyrus (MTG) was measured simultaneously using functional near-infrared spectroscopy. **Results:** Mid-frequency narrowband noise significantly improved postural stability, as evidenced by reductions in sway length, ellipse surface, and delta Y compared to the high-frequency and no-sound conditions (*p* < 0.05). In contrast, high-frequency narrowband noise consistently produced the greatest postural instability, with significantly larger ellipse surface and delta Y values (*p* < 0.05). Regarding cortical activation, high-frequency stimulation induced significantly greater bilateral activation in the PMC compared to low-frequency stimulation, while all auditory conditions elicited widespread activation across PMC, STG, and MTG. **Conclusions:** These findings suggest that the frequency characteristics of auditory stimulation exert differential neurophysiological effects on balance control. Mid-frequency narrowband noise may enhance static balance by facilitating sensorimotor integration, whereas high-frequency narrowband noise may induce PMC hyperactivation, potentially contributing to postural instability.

## 1. Introduction

Balance ability is an essential capacity for performing daily activities independently, playing a critical role in maintaining postural alignment and preventing falls [1,2,3]. Proper balance control becomes even more crucial when performing multiple tasks simultaneously rather than single movements, and it is directly linked to improvements in quality of life [4]. While previous studies have primarily explored the roles of visual, vestibular, and somatosensory inputs in maintaining balance, there has recently been growing interest in the potential contribution of auditory information [5]. Auditory input, including differences in processing time and intensity between both ears, is involved in providing spatial location information and can serve as additional sensory feedback for postural control [6,7]. In fact, studies have reported that auditory stimulation can enhance balance ability, with changes in postural regulation depending on the type of auditory input presented [8]. Research comparing the effects of various sounds, such as broadband acoustic noise, narrow-band noise, and pure tones, has suggested that differences in frequency bandwidth may significantly influence postural control [9].

Broadband acoustic noise, characterized by a continuous and uniform spectrum ranging from 20 to 20,000 Hz, has been widely utilized in auditory stimulation studies [10]. Previous research has shown that replacing music with broadband acoustic noise can improve cognitive performance, and it has been reported to exert positive effects on cortical activation and motor control [9,11]. Even in healthy adults, broadband acoustic noise has been shown to stimulate somatosensory input, promote motivation, and reduce postural sway [12]. Furthermore, broadband acoustic noise can contribute to fall prevention among individuals whose balance is compromised by visual, vestibular, or somatosensory deficits, with particularly pronounced effects in those with peripheral sensory impairments [12,13]. While some studies suggest that middle- and high-frequency noise has the potential to reduce postural instability [14], other investigations, such as Park et al. [15], present conflicting empirical evidence, demonstrating that high-frequency auditory stimulation significantly exacerbates postural sway. To reconcile these divergent findings, it is essential to consider how varying task difficulties and baseline cognitive loads modulate sensorimotor integration.

The effects of broadband acoustic noise on postural instability have been investigated using neurophysiological and neuroimaging methods, such as EEG and fMRI, to examine auditory stimulation-related changes in brain activity [16,17]. Auditory information is transmitted from the primary auditory cortex to the temporal lobe, and the middle and anterior regions of the temporal lobe demonstrate distinct responses to auditory stimulus recognition [18]. Additionally, the premotor cortex, which plays a central role in balance and postural regulation, has been reported to participate in modulating brain activation and promoting postural control in response to broadband acoustic noise stimulation [17]. These mechanisms are interpreted as being based on stochastic resonance, which lowers the sensory threshold of the nervous system and increases sensitivity to input signals.

Therefore, the purpose of this study is to identify the effects of presenting narrowband noise of three different frequency bands as auditory stimulation in a sound-shielded environment on static balance ability and cortical activation during tandem stance performed with eyes closed. However, investigating these underlying mechanisms via functional near-infrared spectroscopy (fNIRS) necessitates rigorous methodological controls. According to modern consensus guidelines for fNIRS publications, measuring cortical activation during physical tasks requires strict consideration of systemic extra-cerebral hemodynamics [19]. Because physical effort and sensory processing induce systemic autonomic arousal, cortical signal extraction must be carefully interpreted to distinguish true neural responses from superficial blood flow artifacts. This study aims to elucidate the neurophysiological mechanisms through which distinct frequency bands of narrowband noise influence balance control. Rather than proposing clinical or rehabilitative interventions, the primary objective is to establish an exploratory neuro-biomechanical reference model for postural-auditory integration.

## 2. Methods

### 2.1. Participants

This study recruited 20 healthy individuals aged 18 to 35 years. The mean age of the participants was 24.60 ± 2.30 years, with an equal distribution of women (*n* = 10) and men (*n* = 10). The average height was 170.2 ± 9.59 cm, and the mean body weight was 66.95 ± 15.72 kg. The inclusion criteria were as follows: (1) intact neurocognitive, visual, and auditory functions; and (2) normal baseline postural stability, objectively verified by a negative modified Tandem Romberg Test. The exclusion criteria were: (1) history of central or peripheral nervous system disorders; (2) vestibular or neuro-otological diseases, including hearing loss or clinical dizziness; (3) musculoskeletal abnormalities affecting postural control; and (4) previous traumatic brain injury or cranial surgery. All participants received a detailed explanation of the study’s purpose and procedures and voluntarily signed an informed consent form prior to participation. Ethical approval for this study was granted by the Institutional Review Board (IRB No. 2024-02-007-002).

### 2.2. Experimental Procedure

The participants in this study were assessed for cortical activation in the frontal and temporal lobes and for static balance ability under four auditory conditions. The auditory conditions included a no-sound control condition and three frequency-labeled narrowband noise stimulation conditions. The acoustic stimuli utilized in this study were originally sourced from publicly available recordings labeled as ‘frequency-specific narrowband noise.’ However, to ensure physical accuracy, these stimuli are strictly redefined and referred to as narrowband noise throughout this manuscript. The specific stimuli were operationally categorized based on their center frequencies: low-frequency (narrowband noise centered at 500 Hz), mid-frequency (narrowband noise centered at 2000 Hz), and high-frequency (narrowband noise centered at 5000 Hz). In the no-sound condition, participants performed the same experimental task without auditory stimulation. To minimize intensity-dependent physiological confounding effects, all acoustic stimuli were amplitude-normalized across the experimental conditions. During the assessment, the stimuli were delivered bilaterally through noise-canceling earphones to rigorously isolate the auditory input and ensure consistent delivery of the acoustic parameters. The stimuli were delivered through noise-canceling earphones during the experimental task.

Each narrowband noise condition was applied in a randomized order for 3 min and 30 s using noise-canceling earphones. The experiment was conducted in a quiet environment with controlled lighting. Participants performed the tasks barefoot, maintaining a tandem stance position on a balance assessment platform. Prior to each evaluation, they were instructed to cross their arms over their chest, keep their feet in the same position during rest, and place their hands on the chair while keeping their eyes closed. Instructions regarding the start and rest phases were delivered through the initiation of narrowband noise and external auditory cues.

Cortical activation and static balance were assessed using an A-B-A-B-A-B-A block design. In the A phase (rest baseline), participants maintained a stable tandem stance with their eyes closed and hands on the chair for 30 s. This phase served strictly as a hemodynamic recovery period, permitting cortical hemodynamics to return to baseline. In the B phase (task block), participants maintained the identical physical posture for 30 s while specific acoustic conditions (narrowband noise or a no-sound condition) were applied. Crucially, although the physical posture in the no-sound B phase was biomechanically identical to the A phase, it was methodologically defined as an active control (sham) condition. This active control paradigm was implemented to account for task-induced anticipatory arousal and sustained attentional demands, thereby enabling the strict isolation of cortical activation attributable exclusively to the acoustic stimuli. Brain activation and static balance data were recorded simultaneously during each assessment phase. To ensure data accuracy, if a participant failed to maintain the position (e.g., lost balance) during the task, the trial was terminated, and the measurement was repeated.

### 2.3. Measurement Instruments

#### 2.3.1. Functional Near-Infrared Spectroscopy (fNIRS)

To measure changes in cortical activation during static balance maintenance under different narrowband noise conditions, a functional near-infrared spectroscopy system (NIRSport2, NIRx Medical Technologies LLC, Berlin, Germany) was used. The fNIRS measurement employed a sampling rate of 11.6257 Hz and utilized low-intensity light (approximately 0.2 W/cm^2^) to record changes in cerebral oxygenation based on the optical absorption properties of hemoglobin. The montage was designed using the NIRSite software program (version 2.0; NIRx Medical Technologies LLC, Orlando, FL, USA) based on the international standard 10–20 system [20,21].

A total of 38 channels were configured using 14 sources and 13 detectors, recording light intensity at two wavelengths (760 nm and 850 nm). To maintain a 3 cm distance between each source and detector, the NIRStar software (version 15.0; NIRSport2, NIRx Medical Technologies LLC, Berlin, Germany) and the fNIRS Optode Location Decider toolbox (fOLD) were used to determine the positions automatically with anatomical Brodmann area labeling [22,23]. In this study, the regions of interest across the 38 channels were selected to target areas related to auditory and vestibular processing. The specific regions and channels were as follows: Left Pre-Motor and Supplementary Motor Cortex (Channels: 6–9, 21, 23–26, 36), Right Pre-Motor and Supplementary Motor Cortex (Channels: 10–14, 28–30, 32, 33), Left Frontal Eye Fields (Channels: 27, 37), Right Frontal Eye Fields (Channels: 31, 38), Left Superior Temporal Gyrus (Channels: 1, 2, 4, 22), Right Superior Temporal Gyrus (Channels: 15, 18, 19, 34), Left Middle Temporal Gyrus (Channels: 16, 17, 35), and Right Middle Temporal Gyrus (Channels: 3, 5, 20) [17,24].

The fNIRS data were analyzed using nirsLAB version 2019.04 (NIRx Medical Technologies LLC, Berlin, Germany) and NIRS-SPM. Channel signal quality was assessed using the coefficient of variation, and channels with a CV of 15% or less were considered acceptable. Raw light intensity signals were converted into optical density signals, corrected for discontinuities and motion artifacts, and band-pass filtered at 0.001–0.20 Hz with a 15% roll-off width to reduce signal drift and physiological noise. Oxyhemoglobin (HbO) concentration changes were then calculated using the modified Beer–Lambert law.

Task-related HbO activation was analyzed using a general linear model with a canonical hemodynamic response function. The design matrix contrasted rest and task periods, and channel-wise activation was estimated for each participant. Group-level t-maps were generated to identify significant cortical activation patterns, with statistical significance set at *p* < 0.05 and false discovery rate correction applied for multiple comparisons. Beta coefficients were extracted from significant channels as indicators of cortical activation intensity and used for comparisons among auditory stimulation conditions.

#### 2.3.2. Assessment of Static Balance

In this study, static balance under each narrowband noise condition was assessed using the FreeMed pressure measurement system FreeStep (Version 2.0, Sensor Medica, Rome, Italy). The total area of the FreeMed platform was 500 mm × 600 mm, and it was connected to a computer via USB to extract center of pressure (COP) data. The data were calibrated and measured using the manufacturer’s software, FreeStep software (Version 2.02.025, Sensor Medica, Rome, Italy). The analysis of foot pressure was conducted on a stable measurement platform, and the system recorded the following parameters at a sampling frequency of 100 Hz: sway length (the total distance moved by the COP), ellipse surface (the area of the ellipse encompassing 95% of the COP trajectory), Delta X (the maximum COP displacement in the mediolateral direction), Delta Y (the maximum COP displacement in the anteroposterior direction), and average speed (the mean speed of COP movement) [25].

### 2.4. Statistical Analysis

Statistical analyses of the data acquired with the FreeMed system were performed using IBM SPSS Statistics 25.0 (SPSS Inc., Chicago, IL, USA). The Shapiro–Wilk test was used to assess the normality of mean values for the static balance parameters. To examine the effects of narrowband noise frequency bands on static balance, a one-way repeated measures analysis of variance (ANOVA) was conducted. Bonferroni post hoc tests were performed, and the significance level was set at *p* < 0.05.

## 3. Results

### 3.1. Differences in Static Balance with Narrowband Noise Application

**Figure 1:**
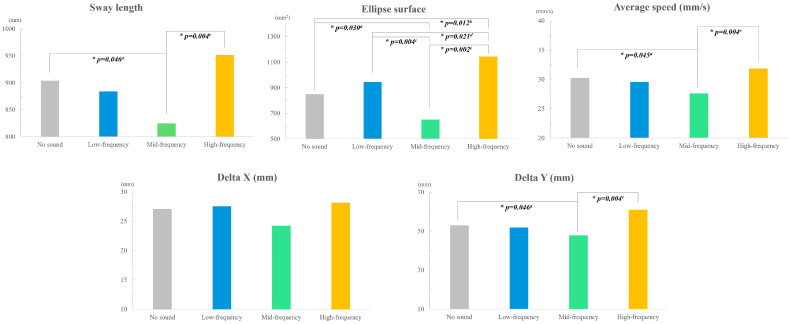
Post hoc comparison of center of pressure (COP) parameters during tandem stance with each narrowband noise frequency. Delta X: maximum lateral (side-to-side) displacement of the COP; Delta Y: maximum anterior–posterior displacement of the COP. Superscript letters indicate significant differences between conditions: **a**: No sound vs. Mid-frequency, **b**: No sound vs. High-frequency, **c**: Low vs. Mid-frequency, **d**: Low vs. High-frequency, **e**: Mid vs. High-frequency. * Indicates statistical significance.

Comparisons of static balance ability based on COP parameters revealed significant differences among conditions in sway length, ellipse surface, average speed, and delta Y (*p* = 0.009), whereas no significant differences were observed in delta X (*p* = 0.053). Sway length was lowest in the mid-frequency condition, showing significant differences compared with the no-sound (*p* = 0.046) and high-frequency conditions (*p* = 0.004). Ellipse surface was also lowest in the mid-frequency condition and differed significantly from both the low- (*p* = 0.004) and high-frequency conditions (*p* = 0.002). In contrast, the high-frequency condition demonstrated the highest ellipse surface value, showing significant differences compared to all other conditions (no: *p* = 0.012, low: *p* = 0.021, mid: *p* = 0.002). Average speed was significantly lower in the mid-frequency condition than in the no-sound (*p =* 0.045) and high-frequency conditions (*p* = 0.004). No other significant differences were found (*p* > 0.05). Regarding delta Y, the mid-frequency condition exhibited the lowest value, which differed significantly from the high-frequency condition (*p* = 0.004). Additionally, the high-frequency condition demonstrated the highest delta Y, showing significant differences compared with the no-sound and mid-frequency conditions (*p* = 0.046) (Figure 1, Table 1).

### 3.2. HbO Dynamics of Cortical Response to Narrowband Noise Frequencies

**Figure 2:**
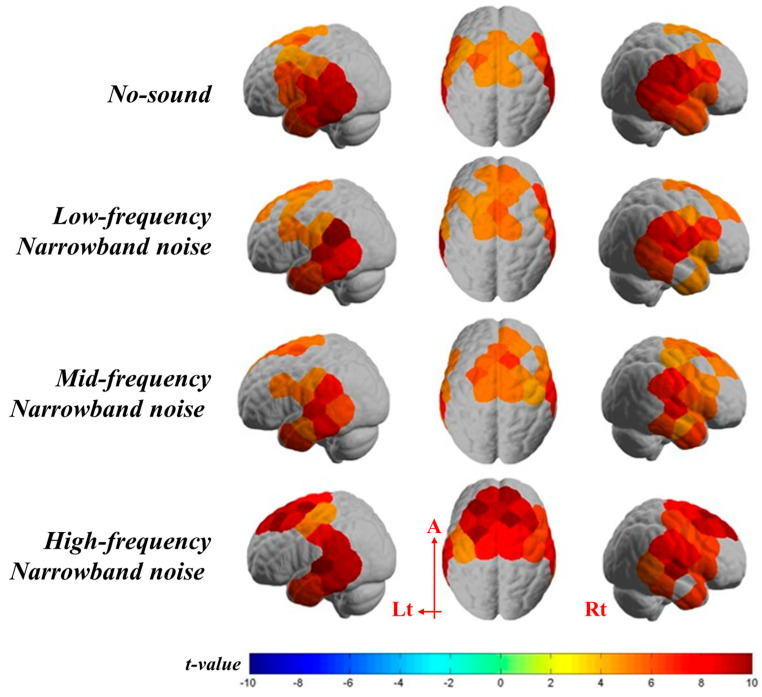
Topographical maps of task-related oxyhemoglobin activation during tandem stance under no-sound, low-frequency, mid-frequency, and high-frequency auditory stimulation conditions. Group-level t-maps were generated using a GLM with a canonical hemodynamic response function and thresholded at *p* < 0.05 with FDR correction for multiple comparisons. Beta coefficients from significant channels indicate the intensity of task-related HbO activation.

During tandem stance without auditory stimulation, significant activation was observed bilaterally in the premotor cortex (PMC), superior temporal gyrus (STG), and middle temporal gyrus (MTG), whereas no significant activation was found in either frontal eye field (FEF). Under the low-frequency condition, significant activation was observed bilaterally in the PMC, FEF, STG, and MTG. During the mid-frequency condition, significant activation was noted bilaterally in the PMC, STG, and MTG and in the right FEF, while no significant activation was detected in the left FEF. In the high-frequency condition, significant activation was observed bilaterally in the PMC, FEF, STG, and MTG (Table 2) (Figure 2).

**Figure 3:**
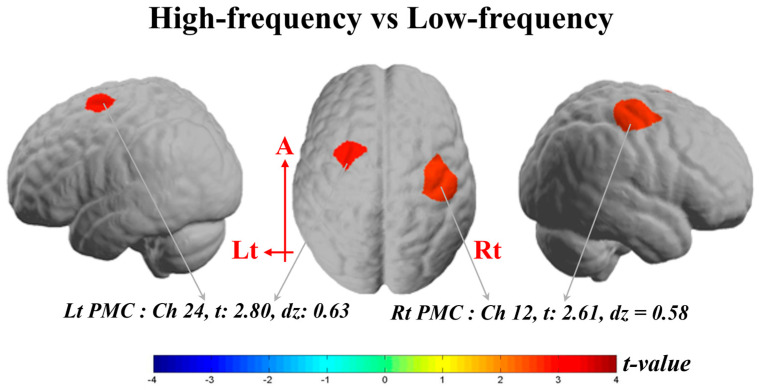
Significant oxyhemoglobin activation differences during tandem stance under high-frequency versus low-frequency auditory stimulation conditions. Group-level GLM t-maps were thresholded at *p* < 0.05 with FDR correction for multiple comparisons. Beta coefficients extracted from significant channels indicate the magnitude of task-related cortical activation.

Comparisons of cortical activation across conditions revealed that significant differences were observed only between the high-frequency and low-frequency conditions. In the high-frequency condition, significantly higher activation was detected bilaterally in the PMC compared to the low-frequency condition (Figure 3). No significant differences in cortical activation were observed among the other sound conditions.

## 4. Discussion

This study was conducted to investigate the effects of narrowband noise of various frequency bands on static postural balance and cortical activation. To this end, adult participants maintained a tandem stance under four conditions, no sound, low-frequency, mid-frequency, and high-frequency, and their static balance indices based on COP (sway length, ellipse surface, delta X, delta Y) and activation of functional brain regions (PMC, FEF, STG, MTG) were measured simultaneously. The results showed that mid-frequency narrowband noise tended to induce the most stable postural control in sway length, ellipse surface, and delta Y, whereas the high-frequency condition consistently produced the greatest instability in these variables. In particular, in the high-frequency condition, significantly higher bilateral PMC activation was observed compared to the low-frequency condition, suggesting an important clue that the frequency characteristics of auditory stimuli are involved in the neurophysiological mechanisms of postural regulation.

In this study, maintaining a tandem stance under mid-frequency narrowband noise led to the most stable outcomes in sway length, ellipse surface, and delta Y. Conversely, the high-frequency narrowband noise condition resulted in the highest instability in these measures. Notably, ellipse surface showed significant differences compared to all other conditions, reflecting the degree of postural sway, as a larger ellipse surface indicates greater variability in COP movement. The absence of significant condition-related differences in mediolateral sway should be interpreted in light of the biomechanical constraints of the tandem stance. Because the heel-to-toe position restricts lateral degrees of freedom, mediolateral COP displacement may have reached a biomechanical ceiling, limiting the extent to which auditory stimulation could further modulate lateral sway [26]. These results suggest that mid-frequency narrowband noise exerts a positive effect on postural stability, whereas high-frequency narrowband noise may act as a destabilizing factor.

A study by Park et al. (2011) [15] investigating the influence of frequency and sound pressure level on static postural stability in healthy young adults reported similar findings [15]. Their main results showed that auditory stimulation at 2000 Hz produced the lowest anteroposterior COP variability, while stimulation at 3000 Hz and 4000 Hz significantly increased COP variability [15]. Additionally, studies on age-related hearing loss have reported that older adults with mid- to high-frequency hearing loss exhibit pronounced declines in balance performance [15]. This suggests that mid- and high-frequency hearing loss commonly observed in older populations may exacerbate balance deterioration associated with visual and vestibular impairments [27]. Overall, mid-frequency narrowband noise stimulation can be considered beneficial for enhancing postural stability, as appropriate levels of auditory input appear to improve the efficiency of motor and sensory postural control systems. In contrast, high-frequency narrowband noise seems to exert relatively adverse effects on balance and motor functions.

Walker et al. (2016) examined physiological responses to high-frequency narrowband noise exposure and observed patterns of sympathetic nervous system activation associated with decreased heart rate variability and mild physiological tension [28]. Consequently, high-frequency narrowband noise stimulation may have negatively influenced postural control and static balance performance, possibly through increased physiological arousal or tension; however, this interpretation should be made with caution because physiological arousal was not directly measured in this study. The discrepancy between studies reporting postural stabilization via high-frequency noise (Siedlecka et al. [14]) and those demonstrating destabilization (Park et al. [15] and the present study) can be explained by the neurocognitive threshold theory. While stochastic resonance facilitates neural signal detection at optimal noise levels, its efficacy is strictly contingent upon the baseline cognitive load and the biomechanical demands of the task. Under low postural demand, high-frequency acoustic input may function as a facilitatory sensory signal. However, maintaining a tandem stance with eyes closed imposes a substantial sensorimotor burden on the central nervous system. The introduction of high-frequency narrowband noise under such high postural demand likely exceeds the optimal neurocognitive threshold. Consequently, the auditory input transitions from a stochastic facilitator to a sensory distractor, disrupting sensorimotor integration and exacerbating mechanical instability.

Regarding cortical activation during static balance, all three narrowband noise stimulations elicited significant activation in the PMC, FEF, STG, and MTG. In contrast, under the no-sound condition, no significant activation of the FEF was observed. The FEF is known as a core region for planning and initiating saccadic eye movements, generating motor signals that direct gaze toward visual targets [29,30]. It also contributes to spatial attention shifts and regulates covert attention, enabling the allocation of attention to specific locations without actual eye movements [30,31]. Accordingly, it can be inferred that low- to high-frequency narrowband noise stimulation increased FEF activation during static balance, whereas maintaining a static stance with eyes closed without auditory input did not require FEF involvement. Previous research investigating the influence of auditory spatial information on the high-level auditory “where” pathway reported widespread activation patterns involving the FEF, HG, PT, IPS, and SPL in response to spatial auditory stimuli. This suggests that the FEF functions not only in visual control but also as part of the auditory spatial map.

High-frequency narrowband noise stimulation, in particular, elicited markedly greater activation of the PMC compared to the low-frequency narrowband noise conditions. The PMC corresponds to Brodmann area 6 and plays a central role in planning, preparing, and executing voluntary movements. Regarding postural control, the PMC is involved in anticipatory postural adjustments and is essential for maintaining body stability during both static and dynamic postures [32,33]. The motor regulation function of the PMC is known to be functionally connected with the prefrontal cortex, primary motor cortex, basal ganglia, and cerebellum [32,34]. Previous studies have reported that certain auditory stimuli activate the PMC [35,36,37]. Recent research that combined auditory stimulation with motor tasks to facilitate motor learning showed that auditory cues produced strong activation of the PMC before movement onset [36]. Previous studies have demonstrated that applying broadband acoustic noise across the entire audible frequency range (20–20,000 Hz) increases motor cortex excitability [17]. Additionally, magnetoencephalography (MEG) studies demonstrated gamma synchrony between the PMC and the auditory cortex during 40 Hz auditory stimulation, suggesting that the PMC helps process patterns and rhythms in auditory information [35,37]. However, excessive cortical activation during postural regulation may often lead to network imbalances in the brain and have negative effects over time. Recent studies in patients with chronic dizziness have shown increased connectivity and hyperactivation in fronto-visual-motor regions during visual stimulation and balance tasks [38]. This has been interpreted as compensatory hyperactivation due to reduced function in specific regions, ultimately exerting a negative impact on postural maintenance [38]. In the present study, the increased bilateral PMC activation observed under high-frequency narrowband noise stimulation likely reflects compensatory recruitment rather than efficient sensorimotor facilitation. However, this finding must be interpreted with strict physiological caution to avoid implying a unidirectional causal sequence where cortical hyperactivation directly induces postural instability. Consistent with bidirectional neurovascular dynamics, it is highly plausible that the high-frequency acoustic stimulus acted as a sensory distractor, primarily perturbing the mechanical equilibrium. Consequently, the observed PMC hyperactivation may represent a secondary, rapid compensatory reaction of the central nervous system attempting to restore physical stability, potentially compounded by task-induced systemic arousal. Therefore, rather than being the definitive root cause of postural failure, this cortical activation should be more accurately framed as a complex, bidirectional correlate of motor instability.

This study has several limitations. First, the sample size was restricted to highly healthy, neurotypical young adults, and the experimental paradigm was strictly confined to a static tandem stance with eyes closed. Consequently, the immediate clinical significance of these findings remains moderate. The neurophysiological responses observed here must be interpreted strictly as an exploratory neuro-biomechanical baseline model, rather than direct evidence for rehabilitative interventions. Second, a critical methodological vulnerability is the absolute omission of short-separation channels in the fNIRS pipeline. Without these channels to regress out systemic extra-cerebral hemodynamics, the recorded cortical activation may be partially confounded by superficial scalp blood flow driven by task-induced autonomic stress. Therefore, the cortical activation results must be interpreted with caution. Third, the functional near-infrared spectroscopy used to measure cortical activation has limitations in spatial resolution and depth sensitivity, making it challenging to analyze subtle activation changes in subcortical structures. Fourth, the auditory stimuli applied in this study were limited to specific narrowband noise conditions. Although the stimuli were amplitude-normalized, strict acoustic parameter controls such as precise decibel calibration using a dosimeter, exact bandwidth mapping, and advanced spatial characteristics were limited due to the reliance on publicly available acoustic sources. Fifth, the fNIRS statistical analysis contains an inherent circularity bias. Because strict a priori anatomical spatial registration was not conducted prior to the analysis, beta coefficients were extracted from channels identified as statistically significant through a data-driven approach, rather than from strictly predefined anatomical Regions of Interest (ROIs). Consequently, the reported effect sizes may be inflated. The fNIRS findings presented herein should therefore be interpreted strictly as exploratory and descriptive data rather than independent confirmatory metrics. Future studies must define ROIs a priori based on standardized anatomical coordinates to ensure strict statistical independence.

## 5. Conclusions

In conclusion, this study investigated the effects of narrowband noise across different frequency bands on static postural balance and cortical activation while participants maintained a tandem stance with their eyes closed. The results demonstrated that mid-frequency narrowband noise produced the most stable postural control, whereas high-frequency noise tended to induce postural instability. Notably, under high-frequency stimulation, significant bilateral hyperactivation of the PMC was observed, suggesting that frequency-labeled narrowband noise stimuli may differentially influence postural stability and cortical activation during static balance control. These findings indicate that auditory stimulation dynamically interacts with vestibular and somatosensory information to modulate postural control networks. However, because this study is methodologically restricted to a highly healthy demographic utilizing a static stance, its immediate clinical significance is moderate. Therefore, this work is most accurately framed as an exploratory neuro-biomechanical baseline model. Future investigations incorporating diverse clinical populations and dynamic balance tasks are imperative to ascertain the actual rehabilitative viability of auditory-based support strategies.

## Figures and Tables

**Table 1:** Comparison of COP parameters during tandem stance with each narrowband noise frequency.

Parameters	No Sound	Low-Frequency	Mid-Frequency	High-Frequency	*p*
Sway length (mm)	903.86 ± 215.18	883.86 ± 180.01	825.04 ± 217.30	951.53 ± 180.05	0.011 *
Ellipse surface (mm^2^)	850.20 ± 416.11	944.87 ± 466.21	651.19 ± 280.98	1144.30 ± 647.40	0.001 *
Average speed (mm/s)	30.27 ± 7.23	29.57 ± 6.04	27.63 ± 7.32	31.88 ± 6.07	0.017 *
Delta X (mm)	27.09 ± 7.36	27.56 ± 7.31	24.24 ± 5.00	28.17 ± 6.56	0.053
Delta Y (mm)	53.06 ± 19.33	51.87 ± 14.69	47.86 ± 18.61	61.01 ± 18.39	0.009 *

Mean value ± standard deviation, * *p* < 0.05.

**Table 2:** Significant HbO-based brain activation regions corresponding to each auditory stimulation condition during tandem stance.

ROI	No Sound	Low-Frequency	Mid-Frequency	High-Frequency
Lt. PMC	6 (5.07, 1.13) 8 (4.59, 1.03) 9 (4.56, 1.02) 21 (7.92, 1.78) 23 (6.17, 1.38) 24 (5.64, 1.26) 25 (4.35, 0.97) 26 (4.70, 1.05) 36 (4.87, 1.09)	6 (4.61, 1.03) 9 (4.89, 1.09) 21 (5.15, 1.15) 23 (5.21, 1.16) 24 (4.65, 1.04) 25 (4.46, 1.00) 26 (4.58, 1.02) 36 (4.91, 1.10)	6 (4.83, 1.08) 9 (5.18, 1.16) 21 (5.58, 1.25) 23 (5.25, 1.17) 24 (5.96, 1.33) 26 (5.52, 1.23) 36 (5.70, 1.27)	6 (7.29, 1.63) 7 (4.38, 0.98) 8 (5.19, 1.16) 9 (7.24, 1.62) 24 (9.38, 2.10) 25 (8.15, 1.82) 26 (8.65, 1.93) 36 (8.88, 1.99)
Rt. PMC	10 (4.60, 1.03) 11 (4.57, 1.02) 13 (9.07, 2.03) 14 (5.82, 1.30) 29 (4.47, 1.00) 30 (4.43, 0.99) 32 (8.00, 1.79) 33 (7.12, 1.59)	10 (5.10, 1.14) 11 (5.90, 1.32) 13 (6.47, 1.45) 14 (4.61, 1.03) 29 (5.08, 1.14) 32 (8.03, 1.80) 33 (6.74, 1.51)	10 (5.23, 1.17) 11 (5.15, 1.15) 12 (4.35, 0.97) 13 (7.50, 1.68) 14 (5.12, 1.14) 28 (4.74, 1.06) 29 (6.58, 1.47) 32 (6.16, 1.38) 33 (4.99, 1.12)	10 (7.62, 1.70) 11 (7.73, 1.73) 12 (6.40, 1.43) 13 (7.79, 1.74) 14 (6.21, 1.39) 28 (7.77, 1.74) 29 (9.72, 2.17) 30 (9.00, 2.01) 32 (7.67, 1.72) 33 (6.55, 1.46)
Lt. STG	1 (8.84, 1.98) 2 (8.18, 1.83) 4 (8.70, 1.95) 22 (5.78, 1.29)	1 (8.05, 1.80) 2 (10.45, 2.34) 4 (9.00, 2.01)	1 (6.19, 1.38) 2 (7.45, 1.67) 4 (8.43, 1.88)	1 (8.89, 1.99) 2 (8.73, 1.95) 4 (9.69, 2.17)
Rt. STG	15 (7.67, 1.72) 18 (8.38, 1.87) 19 (7.37, 1.65) 34 (5.76, 1.29)	15 (7.63, 1.71) 18 (7.67, 1.72) 19 (6.52, 1.46) 34 (4.69, 1.05)	15 (8.07, 1.80) 18 (7.07, 1.58) 34 (5.34, 1.19)	15 (9.07, 2.03) 18 (7.50, 1.68) 19 (5.53, 1.24) 34 (6.47, 1.45)
Lt. MTG	16 (6.75, 1.51) 17 (6.05, 1.35) 35 (5.89, 1.32)	16 (6.07, 1.36) 35 (4.49, 1.00)	16 (6.21, 1.39) 17 (4.41, 0.99) 35 (6.43, 1.44)	16 (6.12, 1.37) 35 (6.27, 1.40)
Rt. MTG	3 (9.58, 2.14) 5 (7.70, 1.72) 20 (6.01, 1.34)	3 (7.91, 1.77) 5 (6.17, 1.38) 20 (6.27, 1.40)	3 (7.86, 1.76) 5 (5.08, 1.14) 20 (6.38, 1.43)	3 (8.81, 1.97) 5 (6.63, 1.48) 20 (6.41, 1.43)
Lt. FEF		37 (4.81, 1.08)		27 (10.06, 2.25) 37 (8.56, 1.91)
Rt. FEF		31 (4.84, 1.08) 38 (5.49, 1.23)	31 (4.76, 1.06) 38 (5.12, 1.14)	31 (8.35, 1.87) 38 (9.44, 2.11)

Channels showing significant changes in fNIRS activation under each auditory stimulation condition. Values are presented as channel number (t-value, Cohen’s dz). Larger Cohen’s dz values indicate greater within-subject effect sizes for blood oxygenation changes. HbO: oxyhemoglobin, ROI: region of interest, PMC: premotor cortex, FEF: frontal eye fields, STG: superior temporal gyrus, MTG: middle temporal gyrus.

## Data Availability

Data available on request due to privacy/ethical restrictions.
